# Self-Reported Complications after Tonsillectomy: Comparison of Responders and Nonresponders to a Mailed Questionnaire

**DOI:** 10.1155/2020/4561858

**Published:** 2020-03-07

**Authors:** Rolf Haye, Liv Kari Døsen, Caryl Gay, Magnus TarAngen, Olga Shiryaeva

**Affiliations:** ^1^Department of Oto-Rhino-Laryngology, Lovisenberg Diaconal Hospital, Oslo 0456, Norway; ^2^Institute of Clinical Medicine, Faculty of Medicine, University of Oslo, Oslo, Norway; ^3^Department of Quality, Lovisenberg Diaconal Hospital, Oslo 0456, Norway

## Abstract

Some studies of tonsillectomy outcomes have low response rates to mailed quality control questionnaires. This study evaluated the effect of nonresponders to mailed questionnaires about posttonsillectomy complications by determining whether mail responders and nonresponders differ. Questionnaires were mailed to patients 3–6 weeks after tonsillectomy to assess postoperative complications, defined as contact with a private practitioner and/or hospital readmission related to postsurgical bleeding, pain, or infection. Nonresponders to the mailed questionnaire were interviewed by telephone 7–11 weeks postoperatively, and responses of mail and telephone responders were compared. Of 818 patients undergoing tonsillectomy during the study period, 66.3% responded by mail, and 29.5% were interviewed by telephone, for a total response rate of 95.7%. The mail response rate was significantly higher among parents of pediatric patients than among adult patients (71.4% versus 58.7%, *p* < 0.001). In the pediatric group, overall complication rates were 65% higher among mail responders than telephone responders (20.9% versus 12.7%, *p*=0.049), likely due to their higher rates of both visits to private practitioners and infection, as there were no differences in rates of pediatric readmission, bleeding, or pain between the responder groups. Among adult patients, mail and telephone responders did not differ with respect to their overall complication rate (40.9% versus 34.1%, *p*=0.226) or their rates of readmission or bleeding. However, similar to the pediatric group, visits to a private practitioner were slightly more common among adult mail responders than telephone responders (30.6% versus 21.1%, *p*=0.065), as were reports of pain (*p*=0.001) and infection (*p*=0.006). Studies relying on mailed questionnaires with low response rates likely overestimate the rate of minor complications handled outside the hospital, but rates of major complications involving readmission are unlikely to be seriously biased by low response rates. Supplementing mailed questionnaires with telephone interviews may increase the validity of surgical outcome studies.

## 1. Introduction

Quality control of tonsillectomy has been ongoing in Sweden for many years [1–3] and has now been introduced in other Nordic countries. Such quality control efforts are important for monitoring surgical outcomes and maintaining optimal patient care. Quality control typically involves mailing questionnaires to patients postoperatively in order to collect information relevant to their surgical outcomes, such as potential complications and ongoing symptoms. However, the findings from these questionnaires are only valid if the patients who respond to them adequately represent the patient population being assessed, in this case, patients undergoing tonsillectomy. Response rates to quality control questionnaires have varied in prior studies, sometimes being as low as 48.1% [2], which can lead to bias due to nonresponses. However, little is known about how nonresponders might differ from those who respond to the mailed questionnaires, and therefore, the extent of nonresponder bias in tonsillectomy quality control is unknown.

To address this gap in the literature, we conducted a study in which patients who did not respond to a mailed quality control questionnaire about potential complications following tonsillectomy were contacted by telephone and asked to respond verbally to the questionnaire. Our primary objective in this study was to determine whether the reported complications of these nonresponders differed from those patients who had responded to the mailed questionnaire. This information is critical to understanding the impact of response rate on the validity of quality control findings. A secondary objective of this study was to determine whether mail response rates differed by patient age group or gender.

## 2. Materials and Methods

The Department of Oto-Rhino-Laryngology at Lovisenberg Diaconal Hospital in Oslo, Norway, performed quality control of tonsillectomy from August 2016 to October 2017 by mailing postoperative questionnaires to all patients. The questionnaire contained a cover letter explaining the purpose of the study, as well as items assessing postoperative complications, complaints, and contact with a health care provider (private practitioner and/or hospital readmission). Together with a prepaid return envelope, these were mailed to adult patients (18 years or older) or the parent of pediatric patients (1–17 years of age) three weeks after surgery. If the questionnaire was not returned within three weeks, the same questionnaire with a return envelope was again mailed to the patient/parent. If no response was received within seven weeks, we contacted the patient/parent by telephone and asked if they would be willing to answer the questionnaire by telephone interview. We limited the number of calls to a maximum of four per patient. The interviewer was a retired surgeon without knowledge of and unknown to the patients.

Only patients who underwent surgery for the indications of recurrent/chronic tonsillitis and/or obstructed breathing were included in the study. All of our tonsillectomies are performed under general anaesthesia. In our practice, the patients have to be a minimum of one year of age and weigh a minimum of 10 kg to be considered for surgery. There is no upper age limit. Most of our surgeries were performed with “cold” instruments. Microdebriders and lasers were not employed. Haemostasis was obtained by compression and supplemented, if necessary, with bipolar diathermy.

The questionnaire contained the following questions:

- Have you/your child, after the tonsillectomy, contacted a private practitioner for any of the following reasons: bleeding, pain, swallowing difficulty, infection, or other reason? Yes or No (for each reason)
- Have you/your child, after the tonsillectomy, been admitted to a hospital for any of the following reasons: bleeding, pain, swallowing difficulty, infection, or other reason? Yes or No (for each reason)
- Have you/your child been operated due to postoperative bleeding? Yes or No
- Was the pre- and postoperative information adequate? Yes or No
- For how long did you/your child use pain medication after surgery: less than one week, one to two weeks, or more than two weeks?
- When were you/your child able to eat a normal diet: within one week, within one to two weeks, or after two weeks?

Unplanned contacts with health care providers (private practitioners and/or hospital readmissions) were used as a proxy measure for overall complication rates following tonsillectomy. Thus, for the assessment of postoperative complications, we determined the number of patients who (1) visited a private practitioner (physician), (2) were admitted to a hospital, and (3) had either contact with a private practitioner or a hospital readmission. For the purpose of this analysis, both private practitioners and hospitals were considered “health care providers”.

Complications were categorized as either minor or major. Minor complications did not require hospital admission and included issues such as bleeding, pain, swallowing difficulties without pain, infection, vomiting, and diarrhoea. Major complications included any issue requiring hospital admission or reoperation. In addition, primary bleeding was defined as bleeding requiring medical attention during the first 24 hours after surgery, while secondary bleeding was defined as bleeding requiring medical attention more than 24 hours after surgery.

The Regional Ethical Committee of South-East Norway determined that this quality control study was exempt from ethical consideration (Ref: 2018/815A).

### 2.1. Statistical Analyses

All analyses were conducted using SPSS version 24 (IBM Corp, Armonk, NY). Descriptive statistics were used to summarize sample characteristics and questionnaire responses. Due to slight differences, the findings for pediatric and adult patients are reported separately. Chi-square tests (or Fisher's Exact Test when any expected cell frequencies were <5) were used for group comparisons on categorical variables, and Mann-Whitney *U* tests were used for group comparisons on ordinal variables. A significance level of *p* < 0.05 was used for all analyses.

## 3. Results

From August 2016 to October 2017, we performed tonsillectomy on 818 patients (440 females and 378 males), of which 507 (62.0%) were tonsillectomies, 273 (33.4%) were adenotonsillectomies, and 38 (4.6%) were tonsillotomies. The indications for surgery were recurrent tonsillitis in 524 (64.1%) and obstructed breathing in 294 (35.9%) patients. The sample included 489 (59.8%) pediatric patients (mean age = 6.3 years, range 1–17) and 329 (40.2%) adult patients (mean age = 28 years, range 18–62). There were more males than females in the pediatric group and more females than males in the adult group (Table 1).

We received mail responses from 542 (66.3%) of the patients/parents between 4 and 8 (mean 5) weeks after surgery. The rest of the patients/parents (n = 276) were contacted by telephone between 7 and 11 (mean = 9) weeks after surgery, of which 241 (87.3%) responded, bringing the total response rate to 783 (95.7%).

The mail response rate was significantly (*p* < 0.001) higher for the pediatric patient group (71.4%) than for the adult group (58.7%), but there were no significant differences in mail response rates between males and females in either age group (pediatric *p*=0.26, adult *p*=0.82).

Adult patients (≥18 years) were more likely than pediatric patients to have contact with a health care provider after surgery (Table 1). This higher overall complication rate among adult patients reflected their higher rates of both visits to a private practitioner and hospital readmission compared to pediatric patients.

As shown in Table 2, contact with a health care provider (private practitioner and/or hospital readmission) was significantly more likely to be reported by mail responders than by telephone responders among the parents of pediatric patients (20.9% versus 12.7%, *p*=0.049), but the difference was not statistically significant among adult patients (40.1% versus 34.1, *p*=0.226). This difference in overall complication rates for pediatric patients was largely due to the nearly double rate of visits to a private practitioner among mail responders compared to telephone responders (16.0% versus 8.5%, *p*=0.041), as hospital readmission rates did not differ between the two responder groups (4.9% versus 4.2%, *p*=0.779). While overall complication rates did not differ between adult mail and telephone responders, there was a nonsignificant trend, similar to that observed among the pediatric patients, for adult mail responders to visit a private practitioner at higher rates than adult telephone responders (30.6% versus 21.1%, *p*=0.065). As with pediatric patients, readmission rates among adult patients did not differ between mail and telephone responders (10.4% versus 13.0%, *p*=0.470).

The main postoperative symptoms reported as the reason for contact with a health care provider are presented by age group in Table 2 for both mail and telephone responders. Some patients reported more than one symptom. Symptoms were generally more likely to be reported by mail responders than telephone responders, with infection being 6 times more likely for the pediatric group (7.2% versus 1.2%, *p*=0.028) and 4.5 times more likely for the adult group (10.9% versus 2.4%, *p*=0.006). Pain was also more likely to be reported by adult mail responders compared to telephone responders (29.5% versus 13.8%, *p*=0.001), but the difference did not reach statistical significance in the pediatric group (11.5% versus 5.9%, *p*=0.084). Only 14 patients in the whole cohort reported visiting a private practitioner for swallowing difficulties without pain and only 13 for some other reason. Due to the small numbers, further analyses were not performed on these outcomes.

Reports of bleeding did not differ between mail and telephone responders in either the pediatric or adult samples. Most bleeding was secondary, as primary bleeding occurred in only five (0.6%) patients. Operative intervention due to secondary bleeding was reported by 7 (1.5%) parents of pediatric patients and by 13 (4.1%) of adult patients, and the rates were similar among those who responded by mail (2.6%, *n* = 14) and by telephone interviews (2.5%, *n* = 6).

The duration of pain medication use and the time interval until the patient resumed a normal diet are presented in Table 3. Although adult patients used pain medication and abstained from eating normally for longer periods than pediatric patients, there were no significant differences between mail and telephone responders on these measures in either the pediatric or adult sample. The pre- and postoperative information provided to patients/parents was considered inadequate by 22 (4.8%) of the parents and 31 (10%) of the adult patients. These rates did not differ significantly between mail and telephone responders in either the pediatric group (4.3% versus 6.1%, *p*=0.449) or the adult group (8.9% versus 11.8%, *p*=0.405).

## 4. Discussion

In this study, we compared complication rates in the three months after tonsillectomy among 542 mail responders and 241 mail nonresponders who were interviewed by telephone. Mail responders were more likely than telephone interviewees to report contact with a health care provider (private practitioner and/or hospital readmission), but this difference was only statistically significant for pediatric patients. Mail responders reported visiting private practitioners more often than nonresponders, mostly due to higher rates of pain and infection, but rates of major complications requiring readmission were similar among mail and telephone responders for both pediatric and adult patients. To our knowledge, this is the only study comparing tonsillectomy quality metrics for responders to mailed questionnaires with those of telephone-interviewed nonresponders.

These findings indicate that quality control efforts that rely solely on mailed questionnaires are likely to overestimate complication rates following tonsillectomy, particularly for minor complications, thereby giving an overly negative impression of what patients and their providers should expect in the postoperative period. The degree to which the complication rates are overestimated is likely affected by the mail response rate, with lower response rates leading to more negatively biased findings than higher response rates.

A prior study [4] found that the most common reason for nonresponse to mailed questionnaires was forgetfulness, but the findings of this study suggest that there may be systematic differences between those who do and do not respond. These findings may reflect increased motivation among patients who have experienced postoperative complications to complete mailed quality control questionnaires, whereas patients without complications may be less motivated to complete them given that they have less to report.

There are also other possible explanations for these findings that should be considered. First, the average time lag between the mail responses and the telephone interviews was four weeks. Although it is unlikely that the patients would have forgotten their complications in this short period, as a postoperative event leading to unplanned contact with a health care provider is not likely to be forgotten, it is possible that the additional time until the telephone interview contributed to reduced reporting of complications. Second, unlike mailed questionnaires, telephone interviews involve verbal contact between the interviewer and the patient. This may lead the patient to minimize their postoperative complications, as such answers may be considered more socially acceptable. However, if this were the case, we would expect that, compared to the telephone responders, the mail responders would also have a higher rate of complaints about not receiving adequate information before and after surgery. Contrary to this, our data indicate that the telephone interviewees (mail nonresponders) tended to be more dissatisfied in this area than the mail responders. Thus, it seems unlikely that the answers we obtained from the telephone interviewees were unduly biased by social acceptability.

In studies of surgical outcomes, it is desirable to obtain as high of a response rate as possible, in order to ensure that the findings accurately represent all patients and not just a systematically biased subgroup of patients. Mailed questionnaires with prepaid return envelopes are often used in such surveys as they are relatively inexpensive and convenient. Given their frequent use, it is important to confirm that the patients who respond to mailed questionnaires do not systematically differ from those who do not; if no meaningful differences exist, information obtained solely from mail responders can be considered reasonably representative of the patient population, assuming that a minimally acceptable level of response has been obtained. It is also important that mailed questionnaires employ strategies for maximizing response rates, thereby minimizing potential for bias. Such strategies have been described in a Cochrane report [5] and have already been implemented at our hospital as part of routinely performed quality control efforts. These methods include limiting the questionnaire to a single one-sided page with only horizontally arranged questions, placing the most important questions at the top of the page, and no open-ended questions. Controversial or embarrassing questions were excluded. These methods likely contributed to the relatively high mail response rate of 66% in this study.

In addition to the high response rate to the mailed questionnaires, it is also important to note that we obtained an extremely high response rate to the telephone interviews among the mail nonresponders. None of the patients or parents refused to answer or complete the telephone interview. Although we did not record the exact number of calls we made to each mail nonresponder in this study, results from an earlier study [4] showed that an average of 1.7 calls were made to each patient in order to complete the telephone interview. In the present study, we found it difficult to reach patients during working hours and were more successful making the calls on weekday evenings or weekend afternoons. Thus, obtaining a high response rate to the telephone calls was less convenient and required more resources than mailed questionnaires.

Nonetheless, the combination of mailed questionnaires and telephone interviews resulted in a total response rate of nearly 96%. This approach was also used by Sarny et al. [6], with similar success. Thus, where resources allow, this combined approach may represent a cost-efficient and effective means of obtaining highly valid and reliable results from quality control of surgical outcomes.

Our secondary objective was to determine whether mail response rates differed by patient age group or gender. The highest mail response rate was obtained for parents of pediatric patients (1–17 years of age). Parents/caregivers possibly feel more grateful to the hospital for the help they obtained for their children than adult patients feel for the same service, and were therefore more likely to respond to the mailed questionnaire. Parents may also have felt more obligated to complete the questionnaire as part of their responsibility for their child's health, which may be a stronger motivator than adult patients' responsibility to themselves for their own health. Consistent with prior studies, there were more females in our adult sample [2, 7–9] and more males in our pediatric sample [2, 8, 10, 11]. However, there were no differences in mail response rates between male and female patients in this study, regardless of their age group.

The tonsillectomy complication rates obtained in this study were consistent with or lower than those of prior studies. The most common complaint after tonsillectomy was pain, regardless of age group. Despite information and adequate prescriptions being delivered to the patient/parent, many patients still find their medication inadequate or troublesome. Most patients turn to their local private practitioner for help and are rarely hospitalised for this problem. In our study, the adult patients, more often than the parents of pediatric patients, contacted a health care provider for help to alleviate pain. A comparison of our findings with those of other studies is shown in Table 4. These data differ according to the type of surgery, the questionnaire response rate, and from which health care institutions the information was obtained. For instance, the low figure in the study by Bhattacharyya [9] may be due to their data being obtained from ambulatory surgery sites, emergency departments, and hospital admissions, whereas the higher rate of health care contact reported in our study may reflect that 75% of the contacts were entirely handled by the patient's local physician. The less common complication of primary postoperative bleeding occurred in 0.6% of our patients, whereas prior studies have reported rates of 0.6% [13], 0.9% [8], 2% [14], and 3.2% [1]. Rates of readmission and surgical intervention for secondary bleeding in our study were favourable comparable to other studies [1–3, 6, 7, 9, 12, 13, 15]. Overall, the complications following tonsillectomy in our department do not seem to exceed those found in reports from other studies, thus increasing the generalizability of our study's findings.

Strengths of this study include the data being collected from a single hospital with a highly standardized quality control protocol, the telephone interviews being performed by a single person unknown to the patients, and the total response rate (mail and telephone combined) being extremely high. However, it is a weakness of the study that the identified differences between the mail and telephone responders are inextricably linked to this study's mail response rate. In settings with a higher or lower mail response rate than the 66% obtained in this study, the differences between responders and nonresponders may vary. Nonetheless, the results of this study suggest that there are at least some differences between mail responders and nonresponders, which could lead to biased estimates of complication rates, even when mail response rates are relatively high. Another weakness of this study was the retrospective data collection of postoperative complications. In future studies, patients will be given a chart prior to the postoperative discharge and will be asked to record unexpected events that may occur during the postoperative period. This prospective approach will aim to minimize potential recall bias.

## 5. Conclusion

In this study, mail responders reported higher rates of minor complications (e.g., pain and infection) that are treated outside of the hospital than mail nonresponders who completed a telephone interview. However, we found no significant differences between mail responders and nonresponders in their rates of major complications requiring readmission or reoperation due to secondary bleeding. These findings indicate that, in studies of tonsillectomy quality control, low response rates to mailed questionnaires may result in negatively biased overestimates of minor complications after tonsillectomy but are unlikely to significantly bias estimates of major complications. Supplementing mailed questionnaires with telephone interviews of mail nonresponders will likely lead to more accurate estimates.

## Figures and Tables

**Table 1:** Participant gender, mail response rates, and complication rates^*∗*^ by age group.

Characteristic/measure	Total sample (*n* = 818)	Pediatric sample (*n* = 489)	Adult sample (*n* = 329)	*p* Value
Gender				<0.001
Male	378 (46.2%)	257 (52.6%)	121 (36.8%)	
Female	440 (53.8%)	232 (47.4%)	208 (63.2%)	

Mail response				<0.001
Responder	542 (66.3%)	349 (71.4%)	193 (58.7%)	
Nonresponder	276 (33.7%)	140 (28.6%)	136 (41.3%)	
	(*n* = 783)^*∗∗*^	(*n* = 467)^*∗∗*^	(*n* = 316)^*∗∗*^	

Any HCP contact^*∗*^	209 (26.7%)	88 (18.8%)	121 (38.3%)	<0.001
PP contact	151 (19.9%)	66 (14.1%)	85 (26.9%)	<0.001
Readmission	58 (7.4%)	22 (4.7%)	36 (11.4%)	<0.001

HCP = health care provider (including either private practitioner or hospital readmission); PP = private practitioner. ^*∗*^Contact with a HCP was used as a proxy measure for overall complication rates. ^*∗∗*^These sample sizes reflect the 95.7% of parents/patients who completed either the mailed questionnaire or telephone interview.

**Table 2:** Comparison of postoperative complication rates and symptoms for mail and telephone responders by age group.

	Total sample (pediatric *n* = 467) (adult *n* = 316)	Mail responders (pediatric *n* = 349) (adult *n* = 193)	Telephone responders (pediatric *n* = 118) (adult *n* = 123)	*p* Value
Any contact with HCP^*∗*^				
Pediatric sample	88 (18.8%)	73 (20.9%)	15 (12.7%)	0.049
Adult sample	121 (38.3%)	79 (40.9%)	42 (34.1%)	0.226

Visit to private practitioner				
Pediatric sample	66 (14.1%)	56 (16.0%)	10 (8.5%)	0.041
Adult sample	85 (26.9%)	59 (30.6%)	26 (21.1%)	0.065

Hospital readmission				
Pediatric sample	22 (4.7%)	17 (4.9%)	5 (4.2%)	0.779
Adult sample	36 (11.4%)	20 (10.4%)	16 (13.0%)	0.470

Postoperative symptoms				
Pediatric sample				
Bleeding	24 (5.1%)	21 (6.0%)	3 (2.5%)	0.139
Pain	47 (10.1%)	40 (11.5%)	7 (5.9%)	0.084
Infection	27 (5.8%)	25 (7.2%)	2 (1.2%)	0.028
Adult sample				
Bleeding	38 (12.0%)	23 (11.9%)	15 (12.2%)	0.941
Pain	74 (23.4%)	57 (29.5%)	17 (13.8%)	0.001
Infection	24 (7.6%)	21 (10.9%)	3 (2.4%)	0.006

HCP = health care provider (private practitioner and/or hospital readmission). ^*∗*^Contact with a HCP was used as a proxy measure for overall complication rates.

**Table 3:** Use of pain medication and time normal diet was resumed for mail and telephone respondents by age group.

	Total sample	Mail responders	Telephone responders	*p* Value
Duration of pain medication use, *n* (%)				
Pediatric sample	(*n* = 459)^*∗*^	(*n* = 341)^*∗*^	(*n* = 118)	0.821
≤1 week	241 (52.5%)	177 (51.9%)	64 (54.2%)	
>1 and ≤2 weeks	202 (44.0%)	154 (45.2%)	48 (40.7%)	
>2 weeks	16 (3.5%)	10 (2.9%)	6 (5.1%)	
Adult sample	(*n* = 307)^*∗*^	(*n* = 186)^*∗*^	(*n* = 121)^*∗*^	0.578
≤1 week	78 (25.4%)	50 (26.9%)	28 (23.1%)	
>1 and ≤2 weeks	186 (60.6%)	110 (59.1%)	76 (62.8%)	
>2 weeks	43 (14.0%)	26 (14.0%)	17 (14.0%)	

Time until normal diet was resumed, *n* (%)				
Pediatric sample	(*n* = 452)^*∗*^	(*n* = 344)^*∗*^	(*n* = 118)	0.556
≤1 week	262 (58.0%)	194 (58.1%)	68 (57.6%)	
>1 and ≤2 weeks	162 (35.8%)	125 (37.4%)	37 (31.4%)	
>2 weeks	28 (6.2%)	15 (4.5%)	13 (11.0%)	
Adult sample	(*n* = 303)^*∗*^	(*n* = 181)^*∗*^	(*n* = 122)^*∗*^	0.090
≤1 week	116 (38.3%)	77 (42.5%)	39 (32.0%)	
>1 and ≤2 weeks	133 (43.9%)	74 (40.9%)	59 (48.4%)	
>2 weeks	54 (17.8%)	30 (16.6%)	24 (19.7%)	

^*∗*^Sample sizes are slightly reduced due to missing data for these items.

**Table 4:** Comparison of postoperative complication rates in recent studies by age group and for the total sample.

Outcome study	Pediatric sample	Adult sample	Total sample
% of patients contacting a HCP for secondary bleeding			
Current study	5.1	12.0	7.9
Harounian et al. [12]	2.8		
Elinder et al. [3]	7.5		
Lowe et al. [13]			3.5
Mueller et al. [14]			7.0
Søderman et al. [1]			9.4
Sarny et al. [6]			24.6
Seshamani et al. [15]		6.3	

% of patients readmitted for secondary bleeding			
Current study	1.5	5.1	4.6
Mueller et al. [14]			4.0
Hallenstål et al. [2]	2.7		5.1
Elinder et al. [3]	5.9		
Battacharyya [9]		5.1	

% of patients operated for secondary bleeding			
Current study	1.5	4.1	2.6
Mueller et al. [14]	2.0		5.0
Elinder et al. [3]	3.3		
Hallenstål et al. [2]			1.7
Søderman et al. [1]			2.7
Sarny et al. [6]			4.7
Brant et al. [7]		3.6	

% of patients contacting a HCP for pain			
Our study	10.0	23.6	15.5
Hallenstål et al. [2]	11.2	31.5	16.8
Elinder et al. [3]	20.4		
Shay et al. [10]	29.6		
Bhattacharyya [9]		2.8	
Seshamani et al. [15]		11.0	11.0

HCP = healthcare provider (private practitioner and/or hospital readmission).

## Data Availability

The data used to support the study can be obtained from Olga Shiryaeva.
